# Supplementary material for: The NERP-4–SNAT2 axis regulates pancreatic β-cell maintenance and function
Source: Nat Commun. 2023 Dec 9;14:8158. doi: 10.1038/s41467-023-43976-8 (PMC10710447; doi:10.1038/s41467-023-43976-8)
Supplement: Supplementary file 1 — Supplementary Information [file 41467_2023_43976_MOESM1_ESM.pdf]

Supplementary Fig. 1

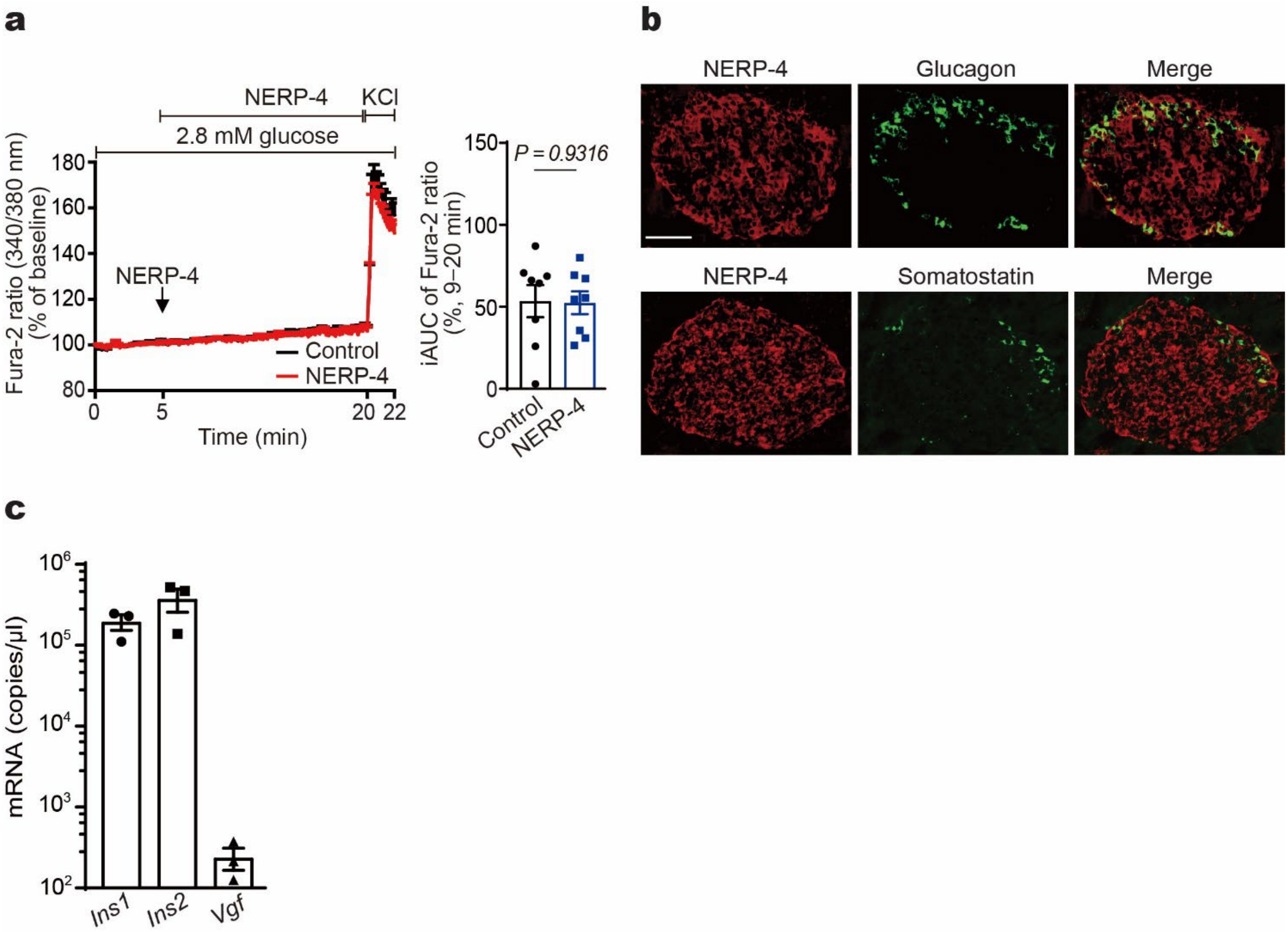

Supplementary Figure 1. NERP-4 is expressed in pancreatic β cells. a, Representative Fura-2-AM ratios in MIN6-K8 cells exposed to NERP-4 under 2.8 mM glucose and average iAUC (9–20 min) of $[Ca^{2+}]_i$ (*n* = 8 cells). **b**, Representative immunofluorescence images of NERP-4, glucagon, and somatostatin in C57BL/6J mouse islets. **c**, *Ins1*, *Ins2*, and *Vgf* mRNA amounts in C57BL/6J mouse islets as determined by digital q-PCR (*n* = 3 biological replicates). Scale bar, 50 μm (**b**). Data are mean ± s.e.m (**a**, **c**). Source data are provided as a Source data file.

Supplementary Fig. 2

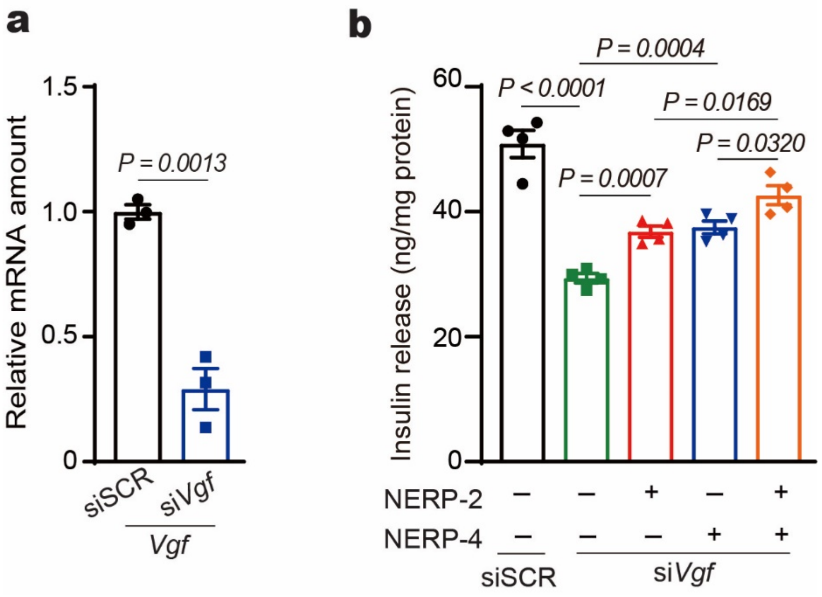

Supplementary Figure 2. **a**, *Vgf* mRNA levels in MIN6-K8 cells transfected with siSCR (n = 3 biological replicates) or si*Vgf* (n = 3 biological replicates). **b**, Additive effect of NERP-4 on NERP-2–induced GSIS in *Vgf* knockdown MIN6-K8 cells. Representative results of two independent experiments. Data are mean ± s.e.m (**a**, **b**). Unpaired two-tailed Student's *t*-test (**a**). One-way ANOVA and Tukey's multiple comparisons test (**b**). Source data are provided as a Source data file.

Supplementary Fig. 3

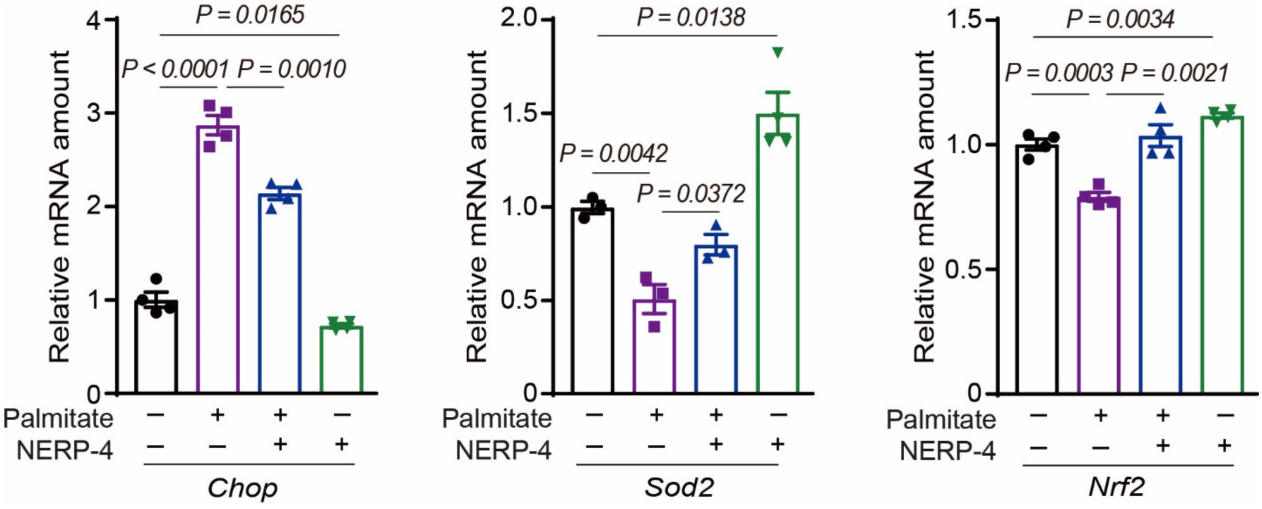

Supplementary Figure 3. NERP-4 was administered at 0, 24 and 48 h to isolated C57BL/6J mouse islets under palmitate. *Chop*, *Sod2,* and *Nrf2* mRNA amounts (*n* = 4, 3, 4 biological replicates). Data are mean ± s.e.m. One-way ANOVA and Tukey's multiple comparisons test. Source data are provided as a Source data file.

Supplementary Fig. 4

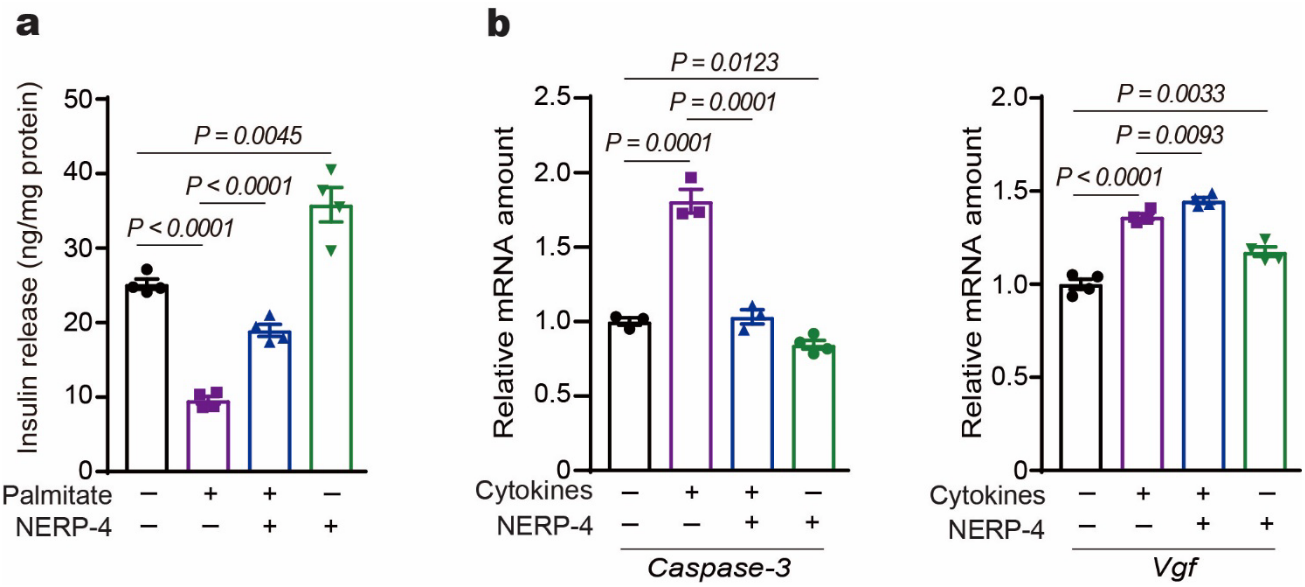

Supplementary Figure 4. a, NERP-4 was administered at 0 and 24 h to MIN6-K8 cells under palmitate. GSIS from MIN6-K8 cells (*n* = 4 biological replicates). **b**, MIN6-K8 cells were treated with a cytokine cocktail and NERP-4 for 24 h. *Caspase-3* (*n* = 3, 4 biological replicates) and *Vgf* (*n* = 4 biological replicates) mRNA amounts. Data are mean ± s.e.m. (**a**, **b**). One-way ANOVA and Tukey's multiple comparisons test (**a**, **b**). Source data are provided as a Source data file.

Supplementary Fig. 5

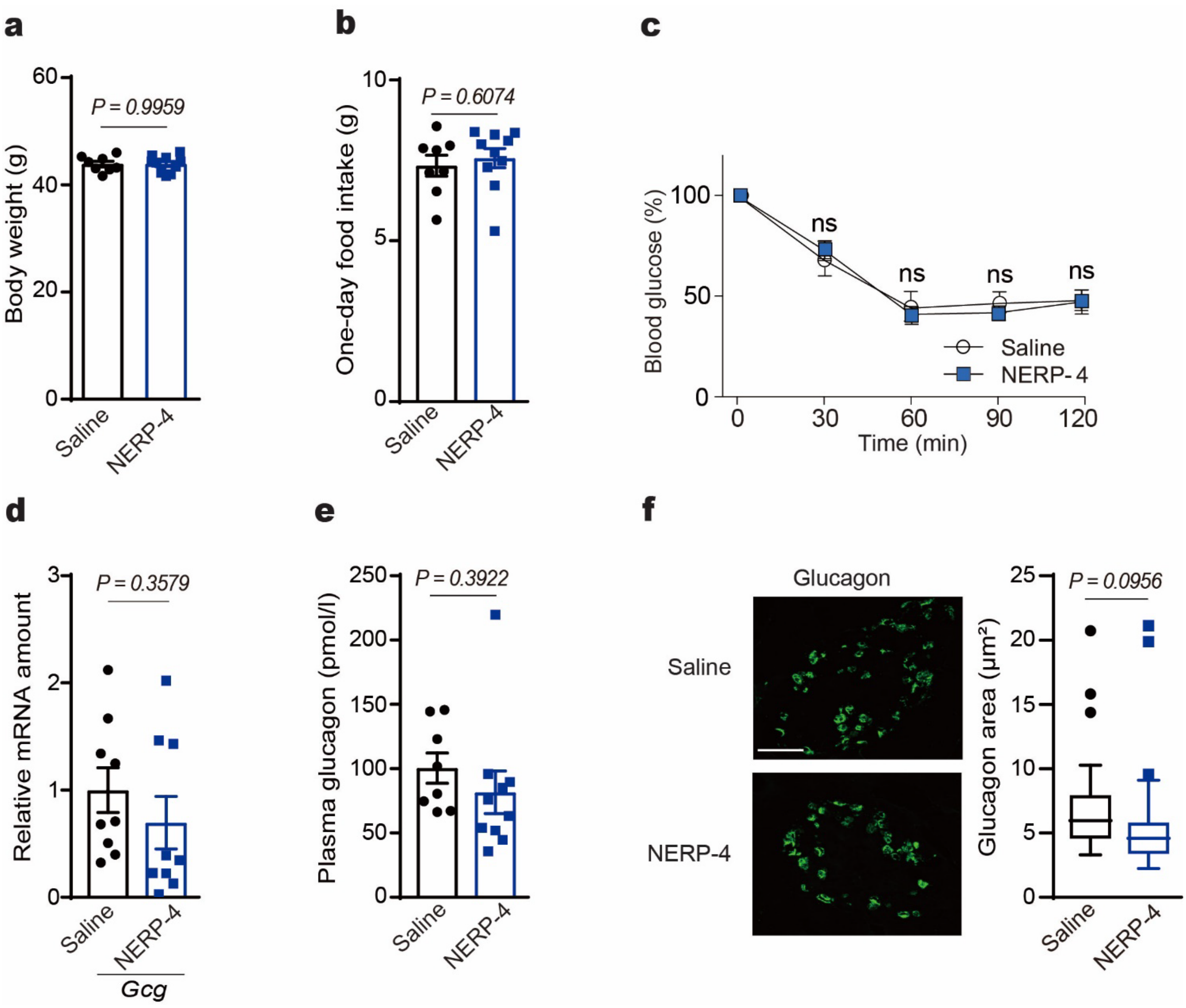

Supplementary Figure 5. *db/db* mice were studied after 2-week administration of NERP-4 or saline. **a**, Body weight (n = 8, 10 biological animals). **b**, One-day food intake (n = 8, 10 biological animals). **c**, Insulin tolerance test (ITT) (n = 10 biological animals). **d**, *Gcg* mRNA amount in pancreatic islets (n = 9 biological animals). **e**, Plasma glucagon levels (n = 8, 10 biological animals). **f**, Representative glucagon immunoreactivity and glucagon-positive area (saline, 43 islets and NERP-4, 37 islets from five mice each). Centre line, median; box edges, first and third quartiles; whiskers, 1.5 times the interquartile range; outliers, individual points (**f**). Results are pooled from two independent experiments (**c**, **d**). Data are mean ± s.e.m (**a**–**e**). Unpaired two-tailed Student's t-test (**a**–**f**). Scale bar,

50 µm (**f**). Source data are provided as a Source data file.

Supplementary Fig. 6

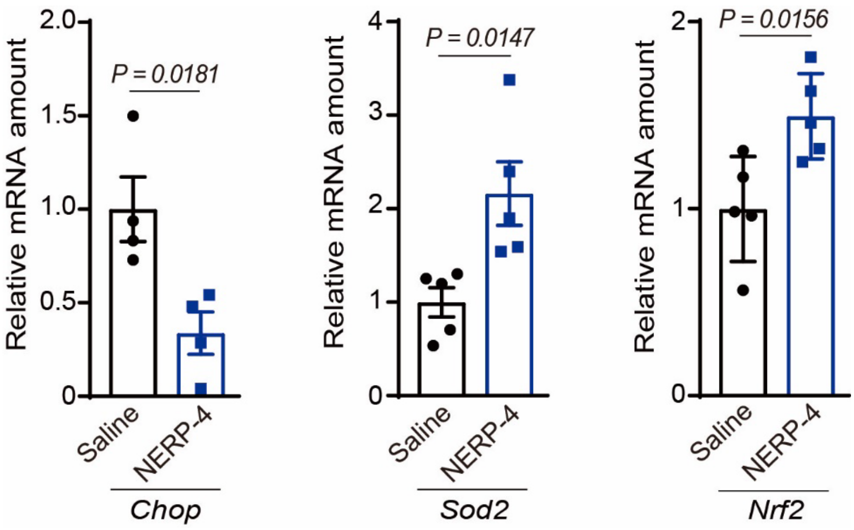

Supplementary Figure 6. *db/db* mice were studied after 2-week administration of NERP-4 or saline. *Chop*, *Sod2*, and *Nrf2* (n = 4, 5 biological replicates) mRNA amounts. Results are representative of three independent experiments. Data are mean ± s.e.m. Unpaired two-sided t-test. Source data are provided as a Source data file.

Supplementary Fig. 7

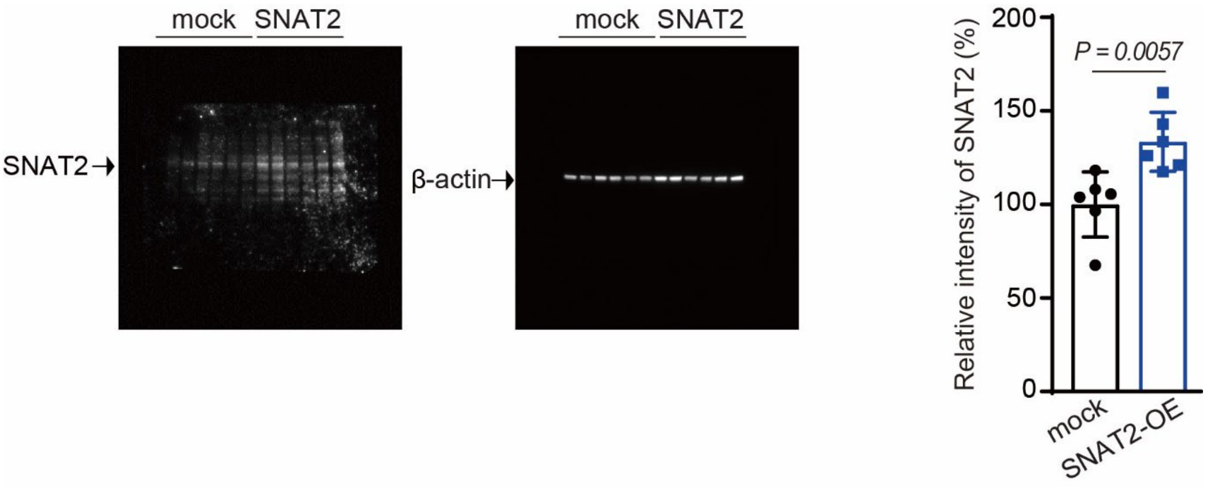

Supplementary Figure 7. Representative western blots for SNAT2 and β-actin in mock or SNAT2-OE HEK293 cells (*n* = 6 biological replicates). The intensity ratio of SNAT2 and β-actin (*n* = 6 biological replicates). Representative results of two independent experiments. Data are mean ± s.e.m. Unpaired two-tailed Student's *t*-test. Source data are provided as a Source data file.

Supplementary Fig. 8

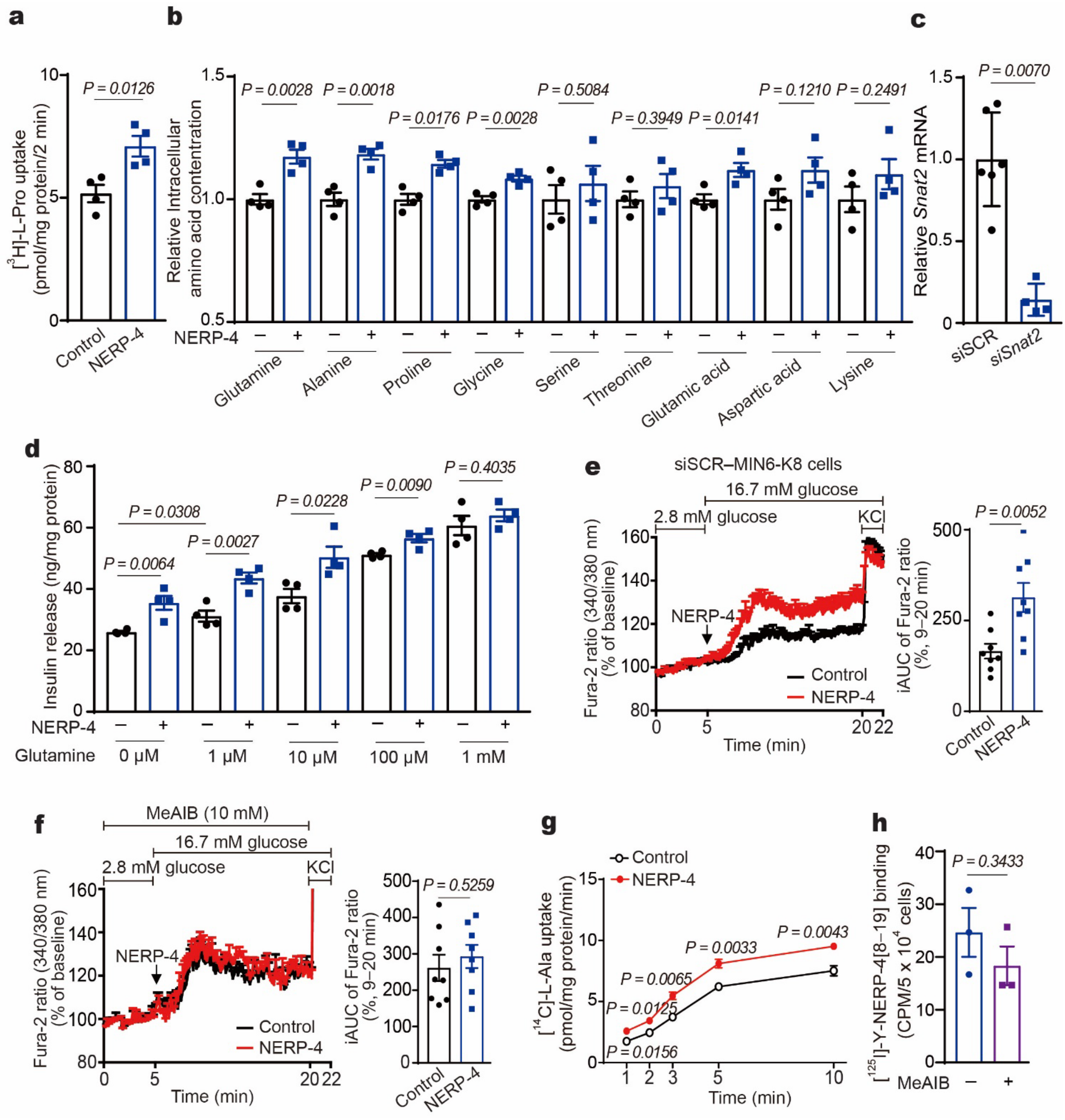

Supplementary Figure 8. **a**, NERP-4–induced [^{3}H]-L-proline uptake in MIN6-K8 cells (n = 4 biological replicates). **b**, Relative intracellular amino acid content in MIN6-K8 cells (n = 4 biological replicates, other amino acids not mentioned were undetected). **c**, *Snat2* mRNA amounts in MIN6-K8

cells transfected with siSCR (n = 6 biological replicates) or si*Snat2* (n = 4 biological replicates). **d**, GSIS in MIN6-K8 cells under different concentrations of glutamine in the presence or absence of NERP-4 (n = 4 biological replicates). **e**, **f**, Representative Fura-2-AM ratios in siSCR– (**e**) or MeAIB-treated (**f**) MIN6-K8 cells in response to NERP-4 (n = 8 cells), and average iAUC (9–20 min) of $[Ca^{2+}]_i$ (n = 8 cells). **g**, Time-dependent $[^{14}C]$-L-alanine uptake in MIN6-K8 cells with or without NERP-4. **h**, Binding of $[^{125}I]$-Y-NERP-4[8–19] to whole cell lysate of MIN6-K8 cells with or without MeAIB (n = 3 biological replicates). Representative results of two independent experiments (**a**–**h**). Data are mean ± s.e.m (**a**–**h**). Unpaired two-sided t-test (**a**, **c**, **e**, **f**, **h**). One-way ANOVA and Tukey's multiple comparisons test (**b**, **d**, **g**). Source data are provided as a Source data file.

Supplementary Fig. 9

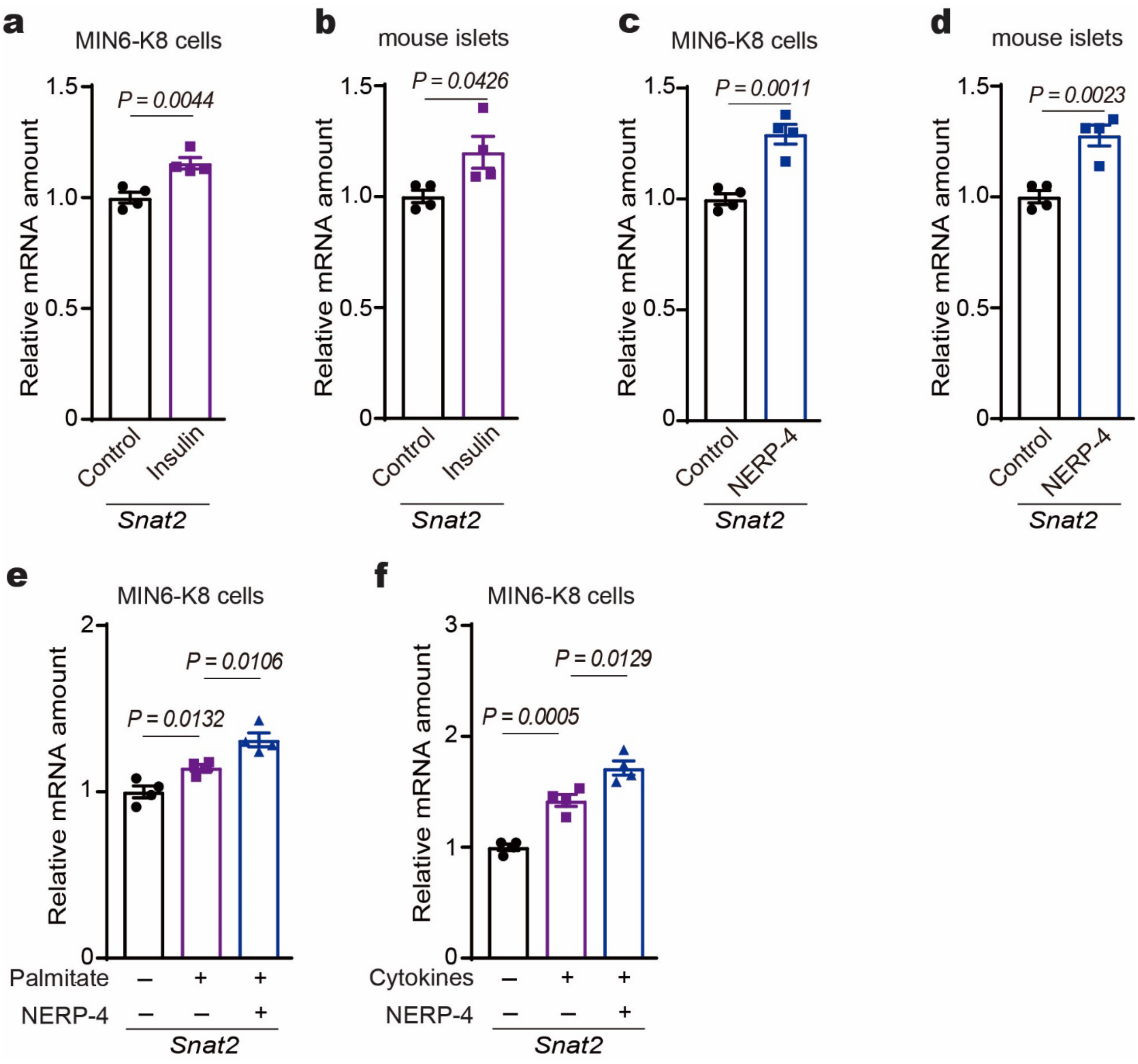

Supplementary Figure 9. a, **b**, MIN6-K8 cells (**a**) and isolated C57BL/6J mouse islets (**b**) were treated with 20 mU/L insulin for 8 h in DMEM containing 4 mM glutamine and 25 mM glucose **(a)** or RPMI-1640 containing 2 mM glutamine and 11 mM glucose (**b**). *Snat2* mRNA amounts in MIN6-K8 cells (**a**) and C57BL/6J mouse islets (**b**) (n = 4 biological replicates). **c**, **d**, MIN6-K8 cells (**c**) and isolated C57BL/6J mouse islets (**d**) were treated with 10^{-10} M NERP-4 for 8 h in DMEM (**c**) or 10^{-8}

M NERP-4 for 8 h in RPMI-1640 (**d**). mRNA amounts of *Snat2* in MIN6-K8 cells (**c**) and C57BL/6J mouse islets (**d**) (n = 4 biological replicates). **e**, **f**, mRNA amounts of *Snat2* in palmitate (**e**)- or cytokines (**f**)-treated MIN6-K8 cells administered 10^{-10} M NERP-4 for 48 or 24 h, respectively (n = 4 biological replicates). Representative results of two independent experiments (**a**–**f**). Data are mean ± s.e.m. (**a**–**f**). Unpaired two-sided *t*-test (**a**–**d**). One-way ANOVA and Tukey's multiple comparisons test (**e**, **f**). Source data are provided as a Source data file.

Supplementary Table 1

Intracellular amino acid contents in MIN6-K8 cells (nmol/mg protein)

	Control	NERP-4	P value
Aspartic acid	35.44 ± 1.49	39.67 ± 1.82	0.1210
Threonine	0.91 ± 0.29	0.96 ± 0.45	0.3949
Serine	1.98 ± 1.14	2.11 ± 1.42	0.5084
Asparagine	ND	ND	
Glutamic acid	27.36 ± 5.70	30.63 ± 7.69	0.0141
Glutamine	1.02 ± 0.22	1.19 ± 0.29	0.0028
Proline	1.93 ± 0.42	2.21 ± 0.31	0.0176
Glycine	30.21 ± 3.86	32.76 ± 3.56	0.0028
Alanine	3.86 ± 1.04	4.57 ± 0.83	0.0018
Valine	ND	ND	
Cystine	ND	ND	
Methionine	ND	ND	
Isoleucine	ND	ND	
Leucine	ND	ND	
Tyrosine	ND	ND	
Phenylalanine	ND	ND	
Histidine	ND	ND	
Tryptophan	ND	ND	
Lysine	1.22 ± 0.63	1.35 ± 0.75	0.2491
Arginine	ND	ND	

Supplementary Table 1. Amino acid contents (pmol/mg protein) in MIN6-K8 cells incubated with HKRB containing 16.7 mM glucose under the presence (n = 4 biological replicates) or absence of NERP-4 (n = 4 biological replicates) for 5 min. Representative results of two independent experiments. Data are mean ± s.e.m. ND, not detected. Unpaired two-sided t-test. Source data are provided as a Source data file.

Supplementary Table 2

Amino acid concentration in the supernatant of MIN6-K8 cells (µM)

	HKRB	2.8 mM glucose	16.7 mM glucose
Aspartic acid	ND	0.32 ± 0.06	0.32 ± 0.05
Threonine	ND	0.78 ± 0.13	0.65 ± 0.09
Serine	ND	0.94 ± 0.08	1.41 ± 0.11
Asparagine	ND	ND	ND
Glutamic acid	ND	ND	ND
Glutamine	ND	0.46 ± 0.02	0.58 ± 0.12
Proline	ND	1.53 ± 0.14	2.05 ± 0.13
Glycine	ND	5.03 ± 0.90	4.11 ± 0.54
Alanine	ND	1.96 ± 0.16	2.10 ± 0.16
Valine	ND	0.61 ± 0.08	0.52 ± 0.09
Cystine	ND	0.48 ± 0.03	0.51 ± 0.06
Methionine	ND	0.23	0.22
Isoleucine	ND	0.46 ± 0.07	0.42 ± 0.08
Leucine	ND	0.90 ± 0.15	0.71 ± 0.13
Tyrosine	ND	0.26 ± 0.04	0.33 ± 0.03
Phenylalanine	ND	0.33 ± 0.05	0.32 ± 0.06
Histidine	ND	ND	ND
Tryptophan	ND	ND	ND
Lysine	ND	0.78 ± 0.12	0.68 ± 0.10
Arginine	ND	0.47	0.42

Supplementary Table 2. Amino acid concentrations (µM) in HKRB buffer (n = 1) and the concentrations of amino acids released from MIN6-K8 cells incubated with HKRB containing 2.8 mM (n =1– 4 biological replicates) or 16.7 mM glucose (n = 1– 6 biological replicates) for 30 min. Data are mean ± s.e.m. ND, not detected. Unpaired two-sided t-test. Source data are provided as a Source data file.

Supplementary Table 3

Real-time PCR primers (TaqMan/Applied Biosciences primers (Thermo Fisher Scientific))

Gene symbol (accession number)	Alias/common name	Primer
Ins1 (NM_008386.3)	Insulin I	Mm01950294_s1
Ins2 (NM_001185083.1)	Insulin II	Mm00731595_gH
Pdx1 (NM_008814.3)	Pancreatic and duodenal homeobox 1	Mm00435565_m1
Iapp (NM_010491.2)	Islet amyloid polypeptide	Mm00439403_m1
Gcg (NM_008101.2)	glucagon	Mm00801714_m1
Pgc1α (NM_008904.2)	Peroxisome proliferative activated receptor, gamma, coactivator 1 alpha	Mm01208835_m1
Drp1 (NM_001025947.2)	Dynamin 1-like	Mm01342903_m1
Park2 (NM_016694.3)	Parkinson disease 2, parkin	Mm01323528_m1
Pink1 (NM_026880.2)	PTEN induced putative kinase 1	Mm00550827_m1
Atp5j2 (NM_020582.2)	ATP synthase, H+ transporting, mitochondrial F0 complex, subunit F2	Mm00834769_g1
Atp5e (NM_025983.3)	ATP synthase, H+ transporting, mitochondrial F1 complex, epsilon subunit	Mm01239887_m1
Casp3 (NM_001284409.1)	Caspase 3	Mm01195085_m1
Gapdh (NM_001289726.1)	Glyceraldehyde-3-phosphate dehydrogenase	Mm99999915_g1
Nrf2 (NM_010902.3)	nuclear factor, erythroid derived 2, like 2	Mm00477784_m1
Vgf (NM_001039385.1)	VGF nerve growth factor inducible	Mm01204485-s1
Chop (NM_001290183.1)	DNA damage inducible transcript 3	Mm01135937_g1
Sod2 (NM_013671.3)	superoxide dismutase 2	Mm01313000_m1
Mfn1 (NM_024200.4)	mitofusin 1	Mm00612599_m1
Snat1 (NM_001166456.1)	solute carrier family 38, member 1	Mm00506391_m1
Snat2 (NM_175121.3)	solute carrier family 38, member 2	Mm00628416_m1
Snat3 (NM_001199217.1)	solute carrier family 38, member 3	Mm01230670_m1
Snat4 (NM_027052.3)	solute carrier family 38, member 4	Mm00459056_m1
Snat5 (NM_172479.3)	solute carrier family 38, member 5	Mm00549967_m1

Supplementary Table 4

Primary Antibodies (Immunocytochemistry/immunohistochemistry)

antibody	Host species/Isotype	Clonality	conjugate	vendor	catalog number	Dilution
Insulin (Ready-to-Use)	Guinea Pig	Polyclonal		DAKO	IS002	Ready-to-Use
Glucagon	Mouse/IgG	Monoclonal		Sigma-Aldrich	G2654	1:4,000
Somatostatin	Rat/IgG	Monoclonal		GeneTex	GTX39061	1:4,000
Ki67 (D3B5)	Rabbit/IgG	Monoclonal		Cell Signaling Technology	9129	1 : 100
NERP-4	Rabbit/IgG	Polyclonal		home made (detail information in METHODS)		1:1,000

Secondary Antibodies (Immunocytochemistry/immunohistochemistry)

antibody	Host species/Isotype	Clonality	conjugate	vendor	catalog number	Dilution
Alexa Fluor 488 goat anti-guinea pig IgG	Goat/IgG	Polyclonal	Alexa Fluor 488	Invitrogen	A11073	1:1,000
Alexa Fluor 488 donkey anti-mouse IgG	Donkey/IgG	Polyclonal	Alexa Fluor 488	Invitrogen	A21202	1:1,000
Alexa Fluor 488 goat anti-rat IgG	Goat/IgG	Polyclonal	Alexa Fluor 488	Invitrogen	A11006	1:1,000
Alexa Fluor 568 goat anti-rabbit IgG	Goat/IgG	Polyclonal	Alexa Fluor 568	Invitrogen	A11011	1:1,000
Alexa Fluor 568 donkey anti-rabbit IgG	Donkey/IgG	Polyclonal	Alexa Fluor 568	Invitrogen	A10042	1:1,000
Alexa Fluor 488 goat anti-rabbit IgG	Goat/IgG	Polyclonal	Alexa Fluor 488	Invitrogen	A11008	1:1,000
Alexa Fluor 488 donkey anti-rabbit IgG	Donkey/IgG	Polyclonal	Alexa Fluor 488	Invitrogen	A21206	1:1,000

Primary Antibodies (western blotting)

antibody	Host species/Isotype	Clonality	conjugate	vendor	catalog number	Dilution
SNAT2	Rabbit/IgG	Polyclonal		Abcam	ab90677	1:1,000
CHOP	Rabbit/IgG	Monoclonal		Cell Signaling Technology	5554	1:1,000
SOD2	Rabbit/IgG	Monoclonal		Cell Signaling Technology	13194	1:1,000
Nrf2	Rabbit/IgG	Polyclonal		Proteintech	16396-1-AP	1:3,000
Cleaved caspase-3	Rabbit/IgG	Monoclonal		Cell Signaling Technology	9664	1:1,000
Lamin A/C	Rabbit/IgG	Polyclonal		Cell Signaling Technology	2032	1:1,000
beta-actin	Rabbit/IgG	Polyclonal		Sigma-Aldrich	A2066	1:2,000

Secondary Antibodies (western blotting)

antibody	Host species/Isotype	Clonality	conjugate	vendor	catalog number	Dilution
Anti-Rabbit IgG, HRP linked Antibody	Goat/IgG		HRP	Cell Signaling Technology	7074	1:5,000

Function blocking antibodies

antibody	Host species/Isotype	Clonality	conjugate	vendor	catalog number	Dilution
NERP-4	rabbit/IgG	polyclonal		home made (detail information in METHODS)		3 µg/ml
NRS-IgG	rabbit/IgG	polyclonal		WAKO	140-06571	3 µg/ml

Supplementary Table 5

Peptides to examine the specificity of NERP-4 RIA

mouse/human NERP-4
rat NERP-1
rat NERP-2
rat VGF[556–585]
rat VGF[588–617]
angiotensin II
alpha-atrial natriuretic peptide
arginine vasopressin
calcitonin
calcitonin gene-related peptide
met-enkephalin-Arg-Gly-Leu
glucagon
alpha melanocyte-stimulating hormone
melanocyte-concentrating hormone
neurokinin A
neuromedin U
neuropeptide Y
neurotensin
secretin
somatostatin
substance P

Supplementary Table 6

Reagents

Reagents	vendor	country	State/city	catalog number
Human insulin ELISA	Mercodia	Sweden	Uppsala	10-113-01
Morinaga Ultra-sensitive Mouse Insulin ELISA Kit	Morinaga Institute of Biological Science, Inc.	Japan	Yokohama	M1104
NERP-2 peptide	Peptide Institute, Inc.	Japan	Osaka	4444-s
NERP-4 peptide	Peptide Institute, Inc.	Japan	Osaka	custom-made
acetyl-alanylcysteinyl C-terminal dodecapeptide of NERP-4 (acetyl AC-NERP-4[8–19])	Peptide Institute, Inc.	Japan	Osaka	custom-made
C-terminally tyrosyl fragment of NERP-4[8–19]	Peptide Institute, Inc.	Japan	Osaka	custom-made
CellTiter-Glo® Luminescent Cell Viability Assay Kit	Promega	USA	WI, Madison	G7571
Colorimetric/Fluorometric ATP Assay Kit	BioVision	USA	CA, Milpitas	K354-100
Fura 2-AM	Dojindo	Japan	Kumamoto	348-05831
[^{14}C]-L-Glutamine	PerkinElmer	Japan	Yokohama	NEC451
[^{14}C]-L-Alanine	Moravek, Inc.	USA	CA, Brea	MC-466
[^{3}H]-L-Proline	PerkinElmer	Japan	Yokohama	NET483001MC
[^{14}C]-L-methylaminoisobutyric acid	PerkinElmer	Japan	Yokohama	NEC671050UC
α-(Methylamino) isobutyric acid	abcam	USA	MA, Cambridge	ab120552
mouse interferon-γ	Mitltenyi Biotec	Germany	Bergisch Gladbach	130-105-778
mouse TNFα	Mitltenyi Biotec	Germany	Bergisch Gladbach	130-101-688
mouse IL-1β	Mitltenyi Biotec	Germany	Bergisch Gladbach	130-094-053
Cell Counting Kit-8 (CCK-8)	Dojindo	Japan	Kumamoto	CK04
In Situ Cell Death Detection Kit, Fluorescein	Roche Diagnostics K.K.	Japan	Tokyo	12156792910
cAMP Biotrak enzymeimmunoassay system	GE Healthcare	UK	Buckinghamshire	RPN225
NE-PER Nuclear and Cytoplasmic Extraction Reagents	Thermo Scientific	USA	Rockford	YB370765
4',6-Diamidino-2-phenylindole, dihydrochloride (DAPI)	Dojindo	Japan	Kumamoto	D212
Glucagon ELISA Kit	Mercodia	Sweden	Uppsala	10-1281-01
