# Supplementary material for: The NERP-4–SNAT2 axis regulates pancreatic β-cell maintenance and function
Source: Nat Commun. 2023 Dec 9;14:8158. doi: 10.1038/s41467-023-43976-8 (PMC10710447; doi:10.1038/s41467-023-43976-8)
Supplement: Supplementary file 3 — Reporting Summary [file 41467_2023_43976_MOESM3_ESM.pdf]

nature portfolio

Corresponding author(s): MASAMITSU NAKAZATO

Last updated by author(s): Nov 7, 2023

Reporting Summary

Nature Portfolio wishes to improve the reproducibility of the work that we publish. This form provides structure for consistency and transparency in reporting. For further information on Nature Portfolio policies, see our Editorial Policies and the Editorial Policy Checklist.

Statistics

For all statistical analyses, confirm that the following items are present in the figure legend, table legend, main text, or Methods section.

n/a	Confirmed	
☐	☒	The exact sample size (n) for each experimental group/condition, given as a discrete number and unit of measurement
☐	☒	A statement on whether measurements were taken from distinct samples or whether the same sample was measured repeatedly
☐	☒	The statistical test(s) used AND whether they are one- or two-sided<br>*Only common tests should be described solely by name; describe more complex techniques in the Methods section.*
☒	☐	A description of all covariates tested
☐	☒	A description of any assumptions or corrections, such as tests of normality and adjustment for multiple comparisons
☐	☒	A full description of the statistical parameters including central tendency (e.g. means) or other basic estimates (e.g. regression coefficient) AND variation (e.g. standard deviation) or associated estimates of uncertainty (e.g. confidence intervals)
☐	☒	For null hypothesis testing, the test statistic (e.g. F, t, r) with confidence intervals, effect sizes, degrees of freedom and P value noted<br>*Give P values as exact values whenever suitable.*
☒	☐	For Bayesian analysis, information on the choice of priors and Markov chain Monte Carlo settings
☒	☐	For hierarchical and complex designs, identification of the appropriate level for tests and full reporting of outcomes
☒	☐	Estimates of effect sizes (e.g. Cohen's d, Pearson's r), indicating how they were calculated

Our web collection on statistics for biologists contains articles on many of the points above.

Software and code

Policy information about availability of computer code

Data collection	All equipment and software used for data collection are publicly or commercially available. quantitative PCR: Thermal Cycler Dice Real Time System II (Takara Bio); Digital PCR: ProFlex PCR System (Thermo Fisher Scientific) and QuantStudio 3D Analysis Suite Cloud Software (Thermo Fisher Scientific); intracellular calcium influx: digital imaging system (BZ-X700, Keyence); immunohistochemistry images: AX-7 fluorescence microscope (Olympus) and C2 confocal microscope (Nikon); transmission electron microscope images: HT7700 transmission electron microscope (Hitachi); Western blot: Fusion Edge software (Vilber Lourmat); receptor identification: TriCEPS™-based ligand–receptor capture (LRC-TriCEPS, P05201; Dualsystems Biotech); glutamine uptake: Tri-Carb 2810TR (PerkinElmer); amino acid determination: liquid chromatography/mass spectrometry (SRL Inc.); mitochondrial respiration: extracellular Flux Analyzer XFe (Agilent Technologies).
Data analysis	All software used for data collection are publicly or commercially available. All the statistical analyses were performed with GraphPad Prism 7. Image J (NIH V 1.8.0) were used for image analysis.

For manuscripts utilizing custom algorithms or software that are central to the research but not yet described in published literature, software must be made available to editors and reviewers. We strongly encourage code deposition in a community repository (e.g. GitHub). See the Nature Portfolio guidelines for submitting code & software for further information.

Data

Policy information about availability of data

All manuscripts must include a data availability statement. This statement should provide the following information, where applicable:

- Accession codes, unique identifiers, or web links for publicly available datasets
- A description of any restrictions on data availability
- For clinical datasets or third party data, please ensure that the statement adheres to our policy

The authors confirm that all the data supporting the findings of this study are available in the paper and its Extended Data file and Source Data file. The datasets generated during and/or analysed during the current study are available from the corresponding author on reasonable request. Source data are provided in this paper.

Research involving human participants, their data, or biological material

Policy information about studies with human participants or human data. See also policy information about sex, gender (identity/presentation), and sexual orientation and race, ethnicity and racism.

Reporting on sex and gender	Human pancreatic islets were a female, 53-year old donor.
Reporting on race, ethnicity, or other socially relevant groupings	Human pancreatic islets were isolated from a heart-beating cadaveric donor next-of-kin consent for research.
Population characteristics	See above.
Recruitment	Human pancreatic islets were collected by the University Hospital of Lille via the European Consortium for Islet Transplantation human islet distribution program supported by the Juvenile Diabetes Research Foundation.
Ethics oversight	Permissions for research use of human specimens and exportation of human islets were granted by the French Ministry of Higher Education and Research and the Agence de la biomédecine, respectively.

Note that full information on the approval of the study protocol must also be provided in the manuscript.

Field-specific reporting

Please select the one below that is the best fit for your research. If you are not sure, read the appropriate sections before making your selection.

[x] Life sciences [ ] Behavioural & social sciences [ ] Ecological, evolutionary & environmental sciences

For a reference copy of the document with all sections, see nature.com/documents/nr-reporting-summary-flat.pdf

Life sciences study design

All studies must disclose on these points even when the disclosure is negative.

Sample size	No statistical methods were used to pre-determine the sample size. Instead, sample size was chosen based on the previous experience and study (PMID: 31914613, 33857254) to obtain statistical significance and reproducibility.
Data exclusions	We did not exclude any data in this paper.
Replication	Many experiments were repeated multiple times as indicated in the figure legends. Some experiments using human islets were performed once or pooled data because of the shortage of islets.
Randomization	All animal experiments were randomized. For other experiments, including cell experiments, samples were randomly assigned to control and experimental groups by an investigator blinded to subsequent experimental information before performing the corresponding treatment.
Blinding	Investigators were blinded to group allocation during data collection, image quantification and data analysis.

Reporting for specific materials, systems and methods

We require information from authors about some types of materials, experimental systems and methods used in many studies. Here, indicate whether each material, system or method listed is relevant to your study. If you are not sure if a list item applies to your research, read the appropriate section before selecting a response.

Materials & experimental systems

n/a	Involved in the study	
☐	☒	Antibodies
☐	☒	Eukaryotic cell lines
☒	☐	Palaeontology and archaeology
☐	☒	Animals and other organisms
☒	☐	Clinical data
☒	☐	Dual use research of concern
☒	☐	Plants

Methods

n/a	Involved in the study	
☒	☐	ChIP-seq
☒	☐	Flow cytometry
☒	☐	MRI-based neuroimaging

Antibodies

Antibodies used

Antibodies used for immunostaining, neutralization test and western blot in this study are listed in Extended data table 4 of the manuscript with supplier name, catalog number, and dilution used.

Validation

For anti-human NERP-4 antibody, we tested them in Vgf KO mice by immunofluorescence staining, and radioimmunoassay was previously used to valid the specificity of anti-human NERP-4 antibody and anti-rat Vgf antibody as cited in the text. Other antibodies used in this study are commercially available and have been validated by the manufacture, previous studies by other groups, or studies previously published by our lab and cited in the text.
All commercial antibodies have been tested for specificity by their respective suppliers. Antibody validation and validation criteria are available on these websites:
Insulin-https://www.citeab.com/antibodies/3383233-ir002-insulin
Glucagon-https://www.sigmaaldrich.com/JP/ja/product/sigma/g2654
Somatostatin-https://www.genetex.com/Product/Detail/Somatostatin-antibody-YC7/GTX39061
Ki67 (D3B5)-https://www.cellsignal.jp/products/primary-antibodies/ki-67-d3b5-rabbit-mab/9129
SNAT2-https://www.citeab.com/antibodies/779475-ab90677-anti-slc38a2-antibody
CHOP-https://www.cellsignal.jp/products/primary-antibodies/chop-d46f1-rabbit-mab/5554
SOD2-https://www.cellsignal.jp/products/primary-antibodies/sod2-d9v9c-rabbit-mab/13194
Nrf2-https://www.ptglab.co.jp/products/NFE2L2,NRF2-Antibody-16396-1-AP.htm
Cleaved caspase-3-https://www.cellsignal.jp/products/primary-antibodies/cleaved-caspase-3-asp175-5a1e-rabbit-mab/9664
Lamin A/C-https://www.cellsignal.com/products/primary-antibodies/lamin-a-c-antibody/2032
beta-actin-https://www.sigmaaldrich.com/JP/ja/product/sigma/a2066
Anti-Rabbit IgG, HRP linked Antibody-https://www.cellsignal.jp/products/secondary-antibodies/anti-rabbit-igg-hrp-linked-antibody/7074
NRS-IgG-https://labchem-wako.fujifilm.com/jp/product/detail/W01W0114-0657.html

Eukaryotic cell lines

Policy information about cell lines and Sex and Gender in Research

Cell line source(s)

MIN6-K8 cells (provided by Dr. Jun-ichi Miyazaki)
HEK293 cells (ATCC, CRL-1573) were obtained from ATCC.

Authentication

MIN6-K8 cells have been widely used for beta cell related studies in previous publications (e.g. PMID: 31914613, 33857254), and were also authenticated in our lab by insulin secretion studies.
Authentication of HEK293 cells were performed by a Human STR Profiling Cell Authentication Service (ATCC).

Mycoplasma contamination

Cells tested negative for mycoplasma contamination.

Commonly misidentified lines
(See ICLAC register)

No commonly misidentified cell lines were used in the study.

Animals and other research organisms

Policy information about studies involving animals; ARRIVE guidelines recommended for reporting animal research, and Sex and Gender in Research

Laboratory animals

Mice (C57BL/6J background, older than 8 weeks) , db/+ and db/db mice (BKS.Cg-Dock7m+/+LeprdbJ, 8-10 weeks old), Wistar rats (older than 7 weeks) were purchased from Charles River Laboratories; Vgf KO mice were provided by Dr. S.R. Salton (Mount Sinai School of Medicine), and apoaequorin transgenic mice were purchased from Tokyo Research Laboratories (Kyowa Hakko Kogyo Co., Ltd).
Animals were maintained under controlled temperature (21–23 °C) and light (light on: 08:00–20:00) conditions with free access to a standard diet and water.

Wild animals	No wild animals were used in the study.
Reporting on sex	Female human pancreatic islets, male and female mice, male rats were included in the study.
Field-collected samples	No field-collected samples were used in the study.
Ethics oversight	All animal experiments were performed in accordance with the Japanese Physiological Society guidelines for animal care and were approved by the Ethics Committee on Animal Experimentation of the University of Miyazaki.

Note that full information on the approval of the study protocol must also be provided in the manuscript.

Plants

Seed stocks	*Report on the source of all seed stocks or other plant material used. If applicable, state the seed stock centre and catalogue number. If plant specimens were collected from the field, describe the collection location, date and sampling procedures.*
Novel plant genotypes	*Describe the methods by which all novel plant genotypes were produced. This includes those generated by transgenic approaches, gene editing, chemical/radiation-based mutagenesis and hybridization. For transgenic lines, describe the transformation method, the number of independent lines analyzed and the generation upon which experiments were performed. For gene-edited lines, describe the editor used, the endogenous sequence targeted for editing, the targeting guide RNA sequence (if applicable) and how the editor was applied.*
Authentication	*Describe any authentication procedures for each seed stock used or novel genotype generated. Describe any experiments used to assess the effect of a mutation and, where applicable, how potential secondary effects (e.g. second site T-DNA insertions, mosiacism, off-target gene editing) were examined.*
